# Influence of Cyclooxygenase-2 Inhibitors on Kynurenic Acid Production in Rat Brain in Vitro

**DOI:** 10.1007/s12640-018-9952-9

**Published:** 2018-09-03

**Authors:** Izabela Zakrocka, Katarzyna M. Targowska-Duda, Artur Wnorowski, Tomasz Kocki, Krzysztof Jóźwiak, Waldemar A. Turski

**Affiliations:** 1 Department of Experimental and Clinical Pharmacology, Medical University of Lublin, Jaczewskiego 8b, 20-090 Lublin, Poland; 2 Department of Biopharmacy, Medical University of Lublin, Chodźki 4a, 20-093 Lublin, Poland

**Keywords:** Kynurenic acid, Central nervous system, Cyclooxygenase-2, Cyclooxygenase-2 inhibitors, Inflammation

## Abstract

Significant body of evidence suggests that abnormal kynurenic acid (KYNA) level is involved in the pathophysiology of central nervous system disorders. In the brain, KYNA is synthesized from kynurenine (KYN) by kynurenine aminotransferases (KATs), predominantly by KAT II isoenzyme. Blockage of ionotropic glutamate (GLU) receptors is a main cellular effect of KYNA. High KYNA levels have been linked with psychotic symptoms and cognitive dysfunction in animals and humans. As immunological imbalance and impaired glutamatergic neurotransmission are one of the crucial processes in neurological pathologies, we aimed to analyze the effect of anti-inflammatory agents, inhibitors of cyclooxygenase-2 (COX-2): celecoxib, niflumic acid, and parecoxib, on KYNA synthesis and KAT II activity in rat brain in vitro. The influence of COX-2 inhibitors was examined in rat brain cortical slices and on isolated KAT II enzyme. Niflumic acid and parecoxib decreased in a dose-dependent manner KYNA production and KAT II activity in rat brain cortex in vitro, whereas celecoxib was ineffective. Molecular docking results suggested that niflumic acid and parecoxib interact with an active site of KAT II. In conclusion, niflumic acid and parecoxib are dual COX-2 and KAT II inhibitors.

**Electronic supplementary material:**

The online version of this article (10.1007/s12640-018-9952-9) contains supplementary material, which is available to authorized users.

## Introduction

Schizophrenia is a common psychiatric disorder affecting about 1% of the general population (Janoutová et al. 2016). Patients with schizophrenia have shorter life expectancy due to higher cardiovascular and suicidal risk (Piotrowski et al. 2017). Despite many antipsychotic drugs introduced to the market since 1950s, treatment of negative symptoms and cognitive decline during schizophrenia remains important therapeutic challenge (Veerman et al. 2017). Patients’ nonadherence, which may reach up to 55% (Fenton et al. 1997), is one of the most important factors related to schizophrenia treatment failure (Phan 2016). Insufficient antipsychotic drug efficacy explains the importance of searching new agents that may improve schizophrenia course.

Among various theories regarding schizophrenia pathogenesis, the inflammatory system dysregulation hypothesis related to glutamatergic brain dysfunction recently gained some recognition (Girgis et al. 2014). Elevated levels of proinflammatory cytokines have been found in cerebrospinal fluid of schizophrenia patients (Wang and Miller 2017). This was accompanied by impaired blood-brain barrier structure (Schwarz et al. 1998) and changes in postmortem brain tissue (van van Kesteren et al. 2017). Intraperitoneal administration of interleukin (IL)-1β, IL-6, and IL-2 in mice was shown to stimulate dopamine (DA) utilization in prefrontal cortex, leading to DA deficiency, which is connected with negative symptoms in schizophrenia (Zalcman et al. 1994). Murray and O’Connor (2003) reported that cyclooxygenase-2 (COX-2), an enzyme synthesizing prostaglandins involved in inflammatory processes, inhibits long-term potentiation (LTP) in rat dentate gyrus, pointing to COX-2 role in memory and learning. COX-2 was found to be constitutively expressed in both cortical and hippocampal neurons (Yamagata et al. 1993) and being dependent on *N*-methyl-D-aspartate (NMDA) receptor activity (Hewett et al. 2016). The expression of COX-2 could be markedly increased in astroglia and microglia in the presence of inflammatory stimuli (Font-Nieves et al. 2012). COX-2 inhibitors were demonstrated to alleviate memory impairment in diabetic rat model (Yang and Gao 2017), Alzheimer’s disease rat model (Mhillaj et al. 2018), and in humans with first manifestation of schizophrenia (Müller et al. 2010). Neuroprotective effect of COX-2 inhibitor, 5,5-dimethyl-3-(3-fluorophenyl)-4-(4-methylsulphonyl) phenyl-2((5)H)-furanone (DFU) against NMDA-mediated damage of cerebellar granule cells has also been presented (Strauss and Marini 2002).

A link between an impaired inflammatory system and glutamatergic neurotransmission was strengthen after observations that NMDA receptor antagonists, including dizocilpine (MK-801), phencyclidine, and ketamine, may induce schizophrenia symptoms and cognitive impairment (Cadinu et al. 2017). Interestingly, ketamine was reported to induce the expression of IL-6 in neuronal cultures, suggesting a connection between proinflammatory cytokines and psychotomimetic effect of ketamine (Behrens et al. 2008). In line, atypical antipsychotics were shown to be neuroprotective by inhibiting inflammation-induced microglia activation and reactive oxygen species production in cortical neuron-glia cultures (Hu et al. 2012) and by decreasing the level of proinflammatory cytokines in the serum of lipopolysaccharide-treated mice (Sugino et al. 2009). Antagonism towards NMDA receptor of some antipsychotics in rat brain neurons (Barygin et al. 2017) as well as downregulation of NMDA receptor subunit expression in rat thalamus were reported, what may stimulate negative symptoms, and limit these drugs clinical efficacy (Krzystanek et al. 2016).

Kynurenic acid (KYNA), a tryptophan metabolite, is a broad-spectrum antagonist of ionotropic glutamate receptors (Stone and Addae 2002). Kynurenine aminotransferases (KATs) catalyze irreversible conversion of kynurenine (KYN) to KYNA (Nematollahi et al. 2016). Out of four KAT isoenzymes, KAT II is the main isoenzyme responsible for brain KYNA synthesis (Guidetti et al. 2007). On the one hand, KYNA is a well-recognized antiepileptic and neuroprotective agent (Schwarcz et al. 1987). On the other hand, elevated KYNA levels in the brain have been linked with negative schizophrenia symptoms and cognitive decline (Erhardt et al. 2017). Since glutamatergic signaling hypofunction is another important factor in the schizophrenia pathogenesis, the modulation of cortical KYNA production has become a novel target in schizophrenia treatment (Wonodi and Schwarcz 2010).

Because COX-2 inhibitors were shown to have beneficial effects in schizophrenia treatment (Zheng et al. 2017), the goal of our study was to investigate the effect of three COX-2 inhibitors: celecoxib, niflumic acid, and parecoxib on KYNA synthesis and KAT II activity in rat brain cortex in vitro. Additionally, the molecular docking of COX-2 inhibitors to KAT II structure was performed to analyze the possibility of direct KAT II inhibition based on drug structure. Moreover, the analysis of publicly available microarray data concerning the effect of COX-2 inhibitors on KAT-coding genes was conducted.

## Materials and Methods

### Microarray Data Mining

Data on coxibs-dependent modulation of rat *Aadat* gene coding for KAT II enzyme were retrieved from public microarray gene profiling repositories using Perturbation tool of Genevestigator software (Hruz et al. 2008).

### Animals

Experiments were performed on male Wistar rats (Experimental Medicine Center, Medical University, Lublin, Poland), weighing 150–200 g. Animals were kept in standard laboratory conditions with food and water available ad libitum. Experiments were performed between 7 a.m. and 1 p.m. All animals were housed in the laboratory conditions minimum 7 days before procedures were carried out. Experiments presented in this study were accepted by the I Local Ethics Committee for Animal Experiments in Lublin.

### Chemical Substances

Celecoxib, niflumic acid, parecoxib, L-kynurenine (sulfate salt), dimethyl sulfoxide (DMSO), sodium chloride, potassium chloride, magnesium sulfate, calcium chloride, sodium phosphate monobasic, sodium phosphate dibasic, glucose, distilled water, Trizma base, acetic acid, pyridoxal 5′-phosphate, 2-mercaptoethanol, pyruvate, and glutamine were obtained from Sigma-Aldrich. High-performance liquid chromatography (HPLC) reagents were purchased from J.T. Baker Chemicals and from Sigma-Aldrich.

### Evaluation of KYNA Production in Rat Brain In Vitro

Procedures on cortical slices were performed as previously reported by Turski et al. (1989). Rat brains were removed after decapitation from skulls and placed on ice. Brain cortex was immediately dissected from the white matter and cut with a McIlwain tissue chopper (Mickle Laboratory Engineering Co. Ltd., USA). Cortical slices (size 1 mm × 1 mm) were transported into incubation wells (10 slices/well), filled with 1 mL of oxygenated Krebs-Ringer buffer at pH 7.4. The incubation lasted 2 h at 37 °C in the presence of L-KYN (10 μM) and our drugs of interest (10 μM, 100 μM, and 1 mM). Control samples were incubated in the presence of DMSO used as a drug solvent. Six wells were used to analyze each drug concentration. The incubation was terminated by placing the samples into an ice cold bath. After incubation supernatants were centrifuged (15,133 ×*g*, 15 min) and applied to ion exchange resin Dowex 50W+ column. Eluted KYNA was subjected to the HPLC (Thermo Fisher Scientific HPLC system, ESA catecholamine HR-80, 3 μm, C18 reverse-phase column, mobile phase: 250-mM zinc acetate, 25-mM sodium acetate, 5% acetonitrile, pH 6.2, flow rate 1.0 ml/min; fluorescence detector: excitation 344 nm, emission 398 nm) and quantified fluorometrically.

### Evaluation of Kynurenine Aminotransferases Activity in Rat Brain In Vitro

The examination of KAT II activity was conducted according to the method developed by Gramsbergen et al. (1992). In brief, brain cortex was homogenized in 5-mM Tris-acetate buffer (pH 8.0) supplemented with pyridoxal 5′-phosphate (50 μM) and 2-mercaptoethanol (10 mM). Obtained homogenate was centrifuged (15,133 ×*g*, 15 min), and the supernatant was dialyzed for 12 h at 8 °C with the use of cellulose membrane dialysis tubing (average flat width of 10 mm; Sigma-Aldrich) against 4 L of the dialysate buffer, made as described above. Purified enzyme was incubated with tested drugs (10 μM, 100 μM, and 1 mM) in the presence of 2-μM L-KYN (substrate) and L-glutamine (KAT I inhibitor) for 2 h at 37 °C at pH 7.0 in triplicates. The reaction was terminated on ice. Supernatants were centrifuged, and KYNA content was examined chromatographically as cortical slice samples.

### Molecular Docking of COX-2 Inhibitors and Kynurenine to KAT II

**Fig. 1:**
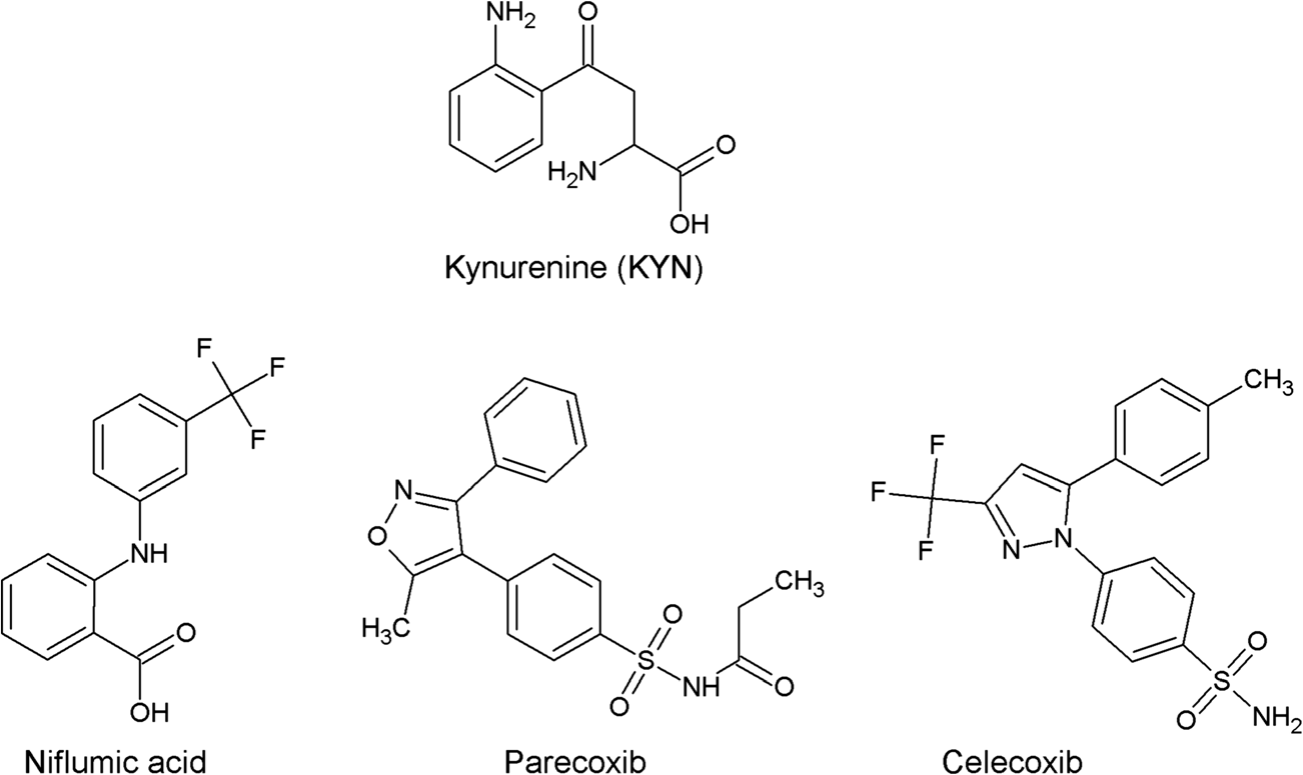
Molecular structures of niflumic acid, parecoxib, celecoxib, and KYN—physiological substrate of KAT II

The available crystal structure of the human KAT II in complex with its substrate L-KYN and co-factor [PMP (4′-deoxy-4′-aminopyridoxal-5′-phosphate)] at 1.95-Å atomic resolution (PDB ID: 2R2N) (Han et al. 2008) was applied to perform the molecular docking as previously described (Zakrocka et al. 2017). More specifically, niflumic acid and parecoxib (for structures see Fig. 1) were imported from the ChEMBL Database and optimized using the semi-empirical method AM1 using Spartan 10 V.1.1.0 (Wavefunction, Inc. Irvine, CA, USA) and then transferred for the subsequent step of molecular docking using Molegro Virtual Docker (v 6.0.0, Molegro ApS, Aarhus, Denmark). The docking space was defined to cover KYN (substrate) and the co-factor (PMP), and the docking simulations were performed using the same setting as previously described (Zakrocka et al. 2017). The correctness of the docking procedure was confirmed by KYN docking to KAT II active site. The lowest energy conformations were selected from each cluster of superposed poses for each studied ligand.

### Statistical Analysis

Mean results are shown as a percentage of control values ± standard deviation (SD). Statistical analysis was conducted using Kruskal-Wallis test followed by Dunn’s multiple comparisons test with GraphPad Prism 6 software. Statistical significance was set at *P* < 0.05.

## Results

### Effect of COX-2 Inhibitors on KAT II Expression in Rat and Human Brain

**Fig. 2:**
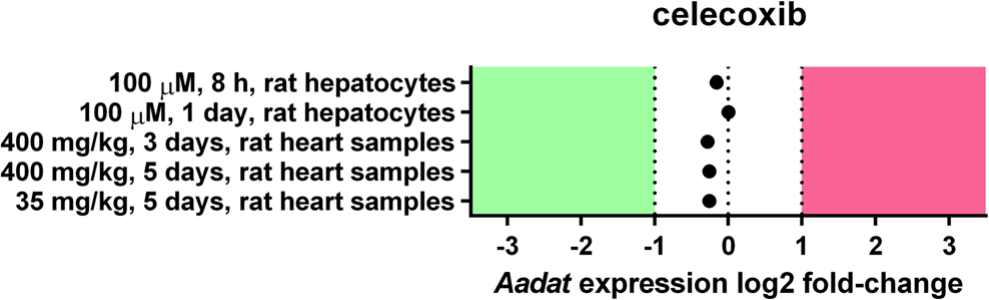
Effect of celecoxib on the expression of *Aadat* (KAT II-coding gene). Data on celecoxib-dependent changes in *Aadat* expression were extracted from publically available gene expression repositories. Neither significant down- nor upregulation of *Aadat* was observed at any experimental condition

Open repositories of microarray experiments were queried for data on coxibs-dependent downregulation of *Aadat* (KAT II-coding gene). Five experiments on celecoxib action towards *Aadat* expression were retrieved. The data originated from rat hepatocytes treated with 100 μM of the drug and heart samples from rats subjected to either 400 or 35 mg/kg of the drug. Expression of *Aadat* was not significantly altered by any of the doses of celecoxib at any tested time-point (Fig. 2). No data on the influence of niflumic acid and parecoxib on *Aadat* expression were available in repositories at the time of the analysis.

### Evaluation of KYNA Production in Brain Cortical Slices In Vitro

**Fig. 3:**
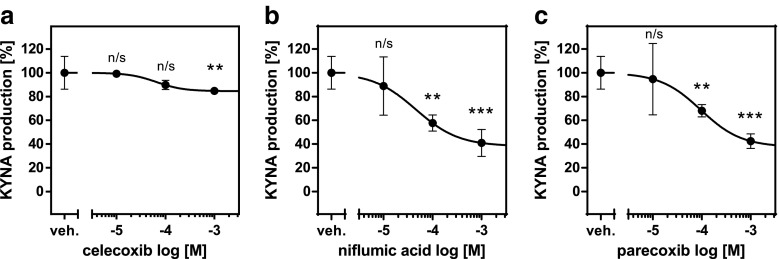
Influence of celecoxib (**a**), niflumic acid (**b**), and parecoxib (**c**) on KYNA production in rat brain cortical slices in vitro. Data are expressed as mean percentage of KYNA production ± SD, *n* = 6, Kruskal-Wallis with Dunn’s post hoc test, ** *P* < 0.01 vs. vehicle control, *** *P* < 0.001 vs. vehicle control

De novo production of KYNA in rat brain slices in vitro under standard conditions was 8.59 ± 0.73 pmol/10 slices/2 h. Celecoxib was inactive at 10- and 100-μM levels and demonstrated only 15% inhibition of KYNA production at 1-mM concentration (Fig. 3a). Niflumic acid decreased KYNA production by 42 and 59% at 100-μM and 1-mM concentration, respectively (Fig. 3b). Parecoxib displayed similar pattern of activity and attenuated KYNA production by 27 and 55% at 100-μM and 1-mM concentration, respectively (Fig. 3c).

### Evaluation of KAT II Activity in Brain Cortical Homogenates In Vitro

**Fig. 4:**
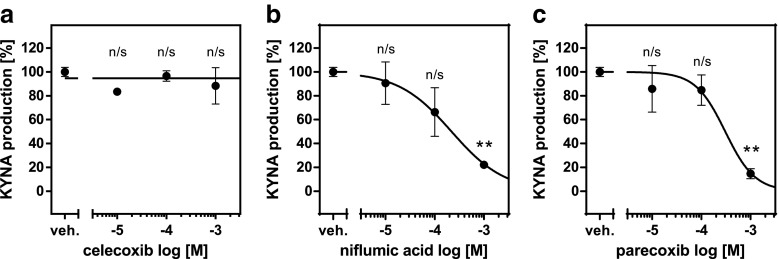
Influence of celecoxib (**a**), niflumic acid (**b**), and parecoxib (**c**) on KAT II activity in rat brain cortex in vitro. Data are expressed as mean percentage of control KYNA production ± SD, *n* = 3, Kruskal-Wallis with Dunn’s post hoc test, ** *P* < 0.01 vs. vehicle control

Mean KYNA production by KAT II under standard conditions was 18.54 ± 0.24 pmol/mL/2 h. Celecoxib displayed no significant inhibitory properties at tested concentrations (Fig. 4a). Niflumic acid and parecoxib (both at 1 mM) lowered KAT II activity in brain cortical homogenates by 78 and 85%, respectively (Fig. 4b, c). At the doses of 10 and 100 μM, niflumic acid and parecoxib did not produce significant inhibition of KAT II activity (Fig. 4b, c).

### Molecular Docking of COX-2 Inhibitors and Kynurenine to KAT II

**Fig. 5:**
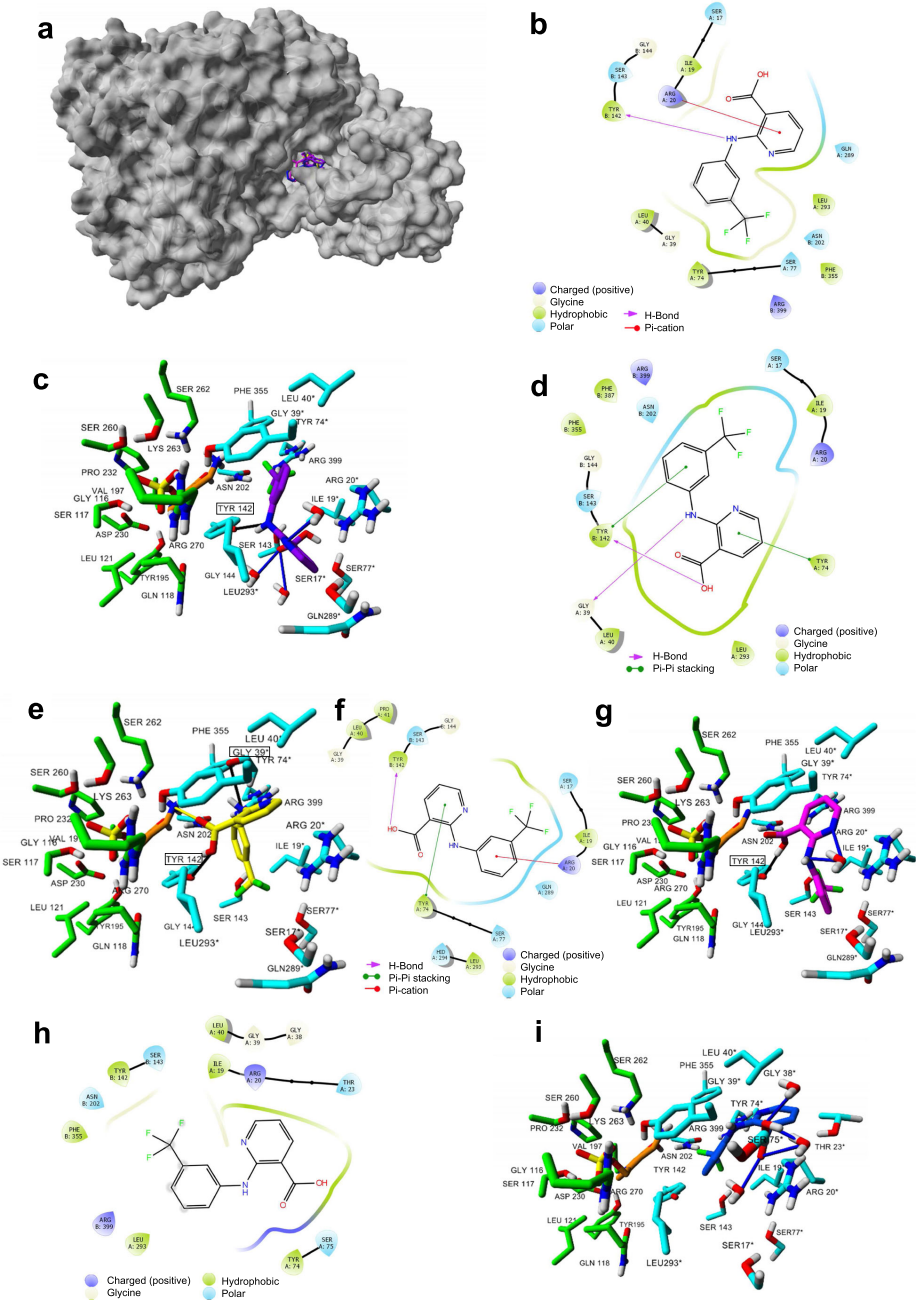
Molecular docking of niflumic acid to the KAT II crystal structure. Four different ligand orientations (**a**) overlap the KYN binding to KAT II active site. **b** 2D and **c** 3D maps for the niflumic acid binding at orientation 1 (purple); **d** 2D and **e** 3D maps for the niflumic acid binding at orientation 2 (yellow); **f** 2D and **g** 3D maps for the niflumic acid binding at orientation 3 (magenta); **h** 2D and **i** 3D maps for the niflumic acid binding at orientation 4 (blue). For 2D maps, each type of interaction determined between niflumic acid—KAT II is included in the respective figure legend. For 3D maps, ligand (shown in purple, yellow, magenta, and blue) and co-factor (shown in orange) are rendered in stick mode, residues involved in ligand and PMP binding are shown in gray and green, respectively. Residues from chain A are labeled with an asterisk to differentiate chain A from chain B residues. Black solid lines represent HBs formed between amino acid residues (marked in black rectangle) and niflumic acid; blue solid lines between ligands and water molecules; while yellow solid lines represent the HBs formed between co-factor and ligand. Oxygen atoms are colored red, nitrogens blue, phosphorus yellow, and chlorine green. Non-polar hydrogen atoms are hidden

Molecular modeling simulations indicate that niflumic acid binds to the active site of KAT II (see Fig. 5 and Table S1 in Supplementary materials) in four possible orientations. In all four orientations, niflumic acid interacts with residues indicated for KYN crystalized with KAT II, including Ile19 (A), Arg20 (A), Gly39 (A), Leu40 (A), Tyr74 (A), Leu293 (A) from one subunit, and Tyr142 (B) and Ser143 (B), from the opposite subunit (see details in Fig. 5). For three orientations (Fig. 5b–e, h–i), additional residues indicated for KYN, including Asn202 (B), Phe355 (B), and Arg399 (B) are also involved in niflumic acid binding. Other residues involved in niflumic acid binding are included in Table S1 (Supplementary materials). However, there are several differences between each orientation. In particular, the hydrogen bond (HB) formed between niflumic acid and co-factor (PMP) is proposed at orientation 2 (Fig. 5e). In addition, different number of HBs are formed at various orientations, including one HB formed between studied ligand and hydroxyl group from Tyr142 (orientations 1 and 3), two between ligand and hydroxyl group from Tyr142 and carbonyl group of Gly39 backbone (orientation 2), while no HBs are indicated for niflumic acid at orientation 4 (Fig. 5). Moreover, water molecules are also involved in niflumic acid binding, including the interaction with KAT II active site at orientations 1 (three molecules), 3 (two molecules), and 4 (three molecules) (Table S1 in Supplementary materials).

**Fig. 6:**
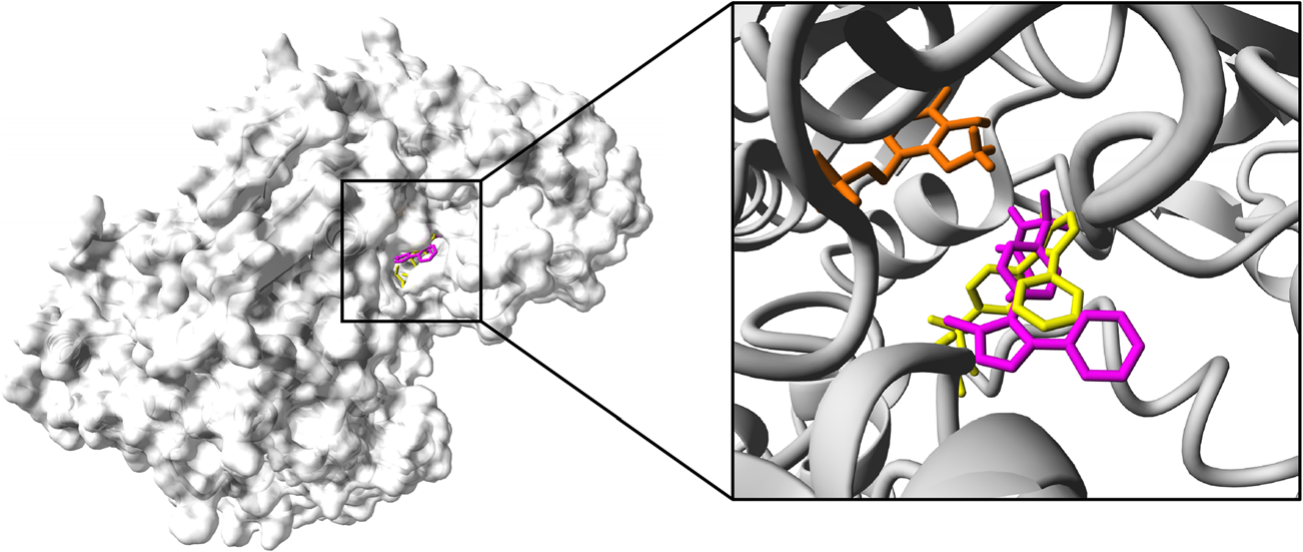
Binding pocket of parecoxib within the KAT II crystal structure. Two ligand orientations overlapping the KYN binding site within the KAT II (Han et al. 2008). Ligand at orientation 1 (yellow) and orientation 2 (magenta) is presented with co-factor; PMP (orange) all rendered in stick mode; KAT II molecular surface is shown in gray. Non-polar hydrogen atoms are hidden

In case of parecoxib, two orientations of the ligand were suggested by the molecular docking (Fig. 6, see Table S1 in Supplementary materials). In both orientations, parecoxib molecule interacts with residues determined for KYN (crystalized with KAT II), including Ile19 (A), Arg20 (A), Gly39 (A), Leu40 (A), Tyr74 (A), Leu293 (A) from one subunit, and Tyr142 (B), Ser143 (B), Asn202 (B), Phe355 (B), Phe387 (B), and Arg399 (B) from the opposite subunit as well as other residues presented in Table S1. Moreover, parecoxib forms HBs with two residues and two water molecules at each orientation. More specifically, two HBs between ligand and guanidinium group from Arg399 and amino group from Asn202 (B) (orientation 1) and two between parecoxib and guanidinium group from Arg399 and Arg20 (orientation 2) (Table S1). Furthermore, HB is also formed between parecoxib and co-factor (PMP) at orientation 2.

## Discussion

In the present study, we showed that COX-2 inhibitors, niflumic acid and parecoxib, decreased KYNA production in rat cortical slices in vitro. What is more, both COX-2 inhibitors lowered KAT II activity, an enzyme that is directly involved in brain KYNA production. Our results are in line with those presented by Schwieler et al. (2005) who reported that parecoxib administered intraperitoneally decreased rat brain KYNA concentration. However, this is the first study in which enzymatic inhibition of KYNA synthesis by COX-2 inhibitors, parecoxib and niflumic acid, is presented. These findings are further supported by the docking of parecoxib and niflumic acid to KAT II active site. In silico results suggest that both ligands interact with residues within the active site of KAT II, mirroring the KYN interactions probably by the competitive mechanism of inhibition. As no data on the ability of investigated COX-2 inhibitors to downregulate KAT II-coding gene, it is possible that, at least in part, parecoxib and niflumic acid-dependent drop in brain KYNA levels occurred due to transcriptional regulation of KAT II.

A growing body of evidence confirms the involvement of inflammatory disturbances in schizophrenia and cognitive decline genesis. The neuroinflammatory theory of schizophrenia originated from studies of Austrian physician Julius Ritter Wagner von Jauregg, who observed that typhus infection alleviated patients’ psychotic symptoms (Wagner 1887). Based on these previous findings, Torrey and Peterson proposed in 1973 that impaired inflammatory responses are key processes in schizophrenia pathogenesis (Torrey and Peterson 1973). COX-2 activation and following prostaglandin production are implicated in various neurological processes (Yui et al. 2015). Transgenic mice with upregulated neuronal COX-2 activity overproducing prostaglandins developed memory impairment and a deficit in aversive behavior (Andreasson et al. 2001). What is more, prostaglandin E_2_ was shown to increase glutamate release in rat spinal cord (Nishihara et al. 1995) and in cultured astrocytes (Bezzi et al. 1998) what may result in neurotoxic effects. Maida et al. (2006) reported that in postmortem studies decreased expression of prostaglandin E_2_ synthase in frontal cortex of patients with schizophrenia was observed.

Interestingly, more evidence suggests a correlation between central and peripheral immunological status in central nervous system disorders. Higher peripheral blood C-reactive protein (CRP) level was correlated with CRP concentration in patients’ cerebrospinal fluid, anhedonia and depressive symptom severity (Felger et al. 2018). Additionally, elevated blood CRP concentration was associated with resistance to treatment in schizophrenia patients (Fond et al. 2018). Patients with first episode of schizophrenia as well as its relapse were reported to have increased serum level of IL-6, tumor necrosis factor α, IL-1β, and interferon-γ and decreased serum concentration of anti-inflammatory interleukin-10 (IL-10) (Müller 2018). Moreover, anti-inflammatory peripheral (Stefanović et al. 2015) and central (Obuchowicz et al. 2017) effects of antipsychotic drugs were presented. Since the brain is no longer an immunologically privileged site the peripheral administration of anti-inflammatory agents can be promising in brain disorders treatment. Interestingly, Schwieler et al. (2005) reported that parecoxib administered intraperitoneally decreased KYNA brain level in rats in contrast to nonselective COX-inhibitors, diclofenac and indomethacin. Suggesting that the effect of parecoxib, a selective COX-2 inhibitor, was related to KAT II tissue inhibition, more KYN available for KYNA synthesis should be expected in peripheral tissues and in the brain, since KYN is well penetrating through blood-brain barrier while KYNA very poor (Sas et al. 2003). As KAT II is responsible for approximately 75% of KYNA synthesis in various brain areas, with less prominent effect in peripheral tissues (Guidetti et al. 1997), a direct KAT II inhibition by parecoxib in rat’s brain appears to be responsible for lower brain KYNA levels. Additionally, Schwieler et al. (2006) reported that parecoxib may impair KYNA formation through decreasing the amount of available KYN. This was supported by other studies revealing that COX-2 inhibitor celecoxib inhibits indoleamine 2,3-dioxygenase (IDO), an enzyme responsible for rate-limiting step of the kynurenine pathway (Basu et al. 2006). In the light of these studies, such complex modulation of KYNA synthesis by COX-2 inhibitors can provide beneficial effects in certain central nervous system disorders.

Indeed, the role of COX-2 inhibitors in memory disorders was already considered. Parecoxib given intraperitoneally for 21 days in ICR mice improved memory performance in the novel object recognition and Y maze tests (Wang et al. 2017). The beneficial effect of parecoxib on short-term memory in rats after splenectomy was also presented (Li et al. 2016). What is more, parecoxib alleviated spatial memory impairment in rats after sevoflurane anesthesia (Gong et al. 2012). Similarly, rofecoxib treatment lasting 7 days in rats receiving excitotoxic agent quisqualic acid significantly attenuated glia activation and a decrease of cortical acetylcholine release (Scali et al. 2003). Cholinergic hypofunction related with memory impairment and astrocyte activation were also lower in rats after 7 days of rofecoxib oral administration (Giovannini et al. 2002). Recently, parecoxib given intravenously was reported to lower the firing activity of dopaminergic neurons in kynurenine 3-monooxygenase knock-out mice, a novel animal model of schizophrenia (Tufvesson-Alm et al. 2018).

Accumulating findings demonstrate clinical efficacy of COX-2 inhibitors in humans. Parecoxib was reported to decrease the incidence of postoperative cognitive dysfunction in patients after total knee arthroplasty (Zhu et al. 2016). Similar results were presented by Tian et al. (2014) and Lu et al. (2017) in elderly patients that received parecoxib before general anesthesia was performed. However, celecoxib failed to improve cognitive performance in randomized controlled trial in patients with Alzheimer’s disease (ADAPT Research Group et al. 2008), whereas according to other researchers the inhibition of cognitive decline after celecoxib administration was observed (Leoutsakos et al. 2012). Additionally, results of celecoxib add-on therapy in schizophrenia patients remain inconclusive. Müller (2017) postulated that adjunct treatment with COX-2 inhibitors may provide better outcome in patients with early stages of schizophrenia and cognitive decline. Nevertheless, most studies present no improvement in schizophrenia patients’ symptoms after celecoxib administration (Rapaport et al. 2005; Sommer et al. 2013). Reported in this study, celecoxib’s limited efficacy in KYNA synthesis inhibition can be in part responsible for drug’s discrepant results on patients with memory impairment or schizophrenia. Thus, further studies are needed to test if other drugs than celecoxib can provide beneficial effects in clinical settings.

It should be emphasized that inhibitors of COX-2 differently affect KAT II activity and these effects seem to be unrelated. Niflumic acid has similar selectivity towards COX-2 compared to celecoxib (COX-1/COX-2 ratio 32) (Grossman et al. 1995). Moreover, Kim et al. (2014) reported that parecoxib does not affect COX-1 and COX-2 activity in cats in vitro. However, our study reveals that niflumic acid and parecoxib are effective inhibitors of KAT II activity and KYNA synthesis in rat brain in vitro, whereas celecoxib is ineffective.

Presented in our study, an inhibition of brain KYNA synthesis is a novel mechanism of examined COX-2 inhibitors, with potential usefulness in memory disorder treatment. Elevated endogenous KYNA levels in rats after receiving KYN intraperitoneally were reported to cause spatial working memory deficits (Chess et al. 2007). Later, Chess et al. (2009) showed that KYN-treated rats had impaired contextual fear memory and their learning was slower compared to control group. Pocivavsek et al. (2012) presented impairment in the passive avoidance test and the Morris water maze test of rats exposed to KYN since prenatal period. Similarly, acute exposure to KYN impaired adult rats’ performance in contextual memory task (Pocivavsek et al. 2017). What is interesting, diet restriction and resulting depletion of KYNA concentration contributed to learning enhancement in *Caenorhabditis elegans* (Vohra et al. 2017). According to studies in humans, an elevated KYNA level in prefrontal cortex is linked with cognitive deficits associated with schizophrenia (Wonodi and Schwarcz 2010). On that account inhibitors of KAT II in the brain were repeatedly investigated as possible novel agents in schizophrenia treatment (Nematollahi et al. 2016; Bortz et al. 2017).

Inhibitory effect of niflumic acid and parecoxib in our in vitro study should be observed after peripheral drug administration. Parecoxib is reported as a hydrosoluble agent (Liu et al. 2016), whereas niflumic acid is an ampholyte (Takács-Novák et al. 2013). Rapid inhibition of brain COX-2 after intravenous parecoxib administration was presented (Mehta et al. 2008). Niflumic acid n.d. passage through the blood brain barrier was also described (https://www.drugbank.ca/drugs/DB04552); however, lipophilic prodrug forms were tested to further improve niflumic acid tissue penetration (el Kihel et al. 1996).

Our study has few limitations. We have presented the effect of COX-2 inhibitors in three different concentrations, up to 1 mM. First, inhibitory effect in our study was achieved at 100-μM parecoxib and niflumic acid concentration. Similarly, 80- and 160-μM parecoxib concentrations were shown to be neuroprotective in rat astrocytes in vitro (Ling et al. 2016). Additionally, niflumic acid up to 160-μM concentration was examined in human-monocyte derived dendritic cells (Svajger et al. 2008). A 1-mM concentration of each examined drug was used in our experiments to analyze if COX-2 inhibitors can saturate KAT II efficiently in a dose-dependent manner. Secondly, parecoxib’s clinical efficacy can be limited in some patients due to reported increased risk of cardiovascular events (Aldington et al. 2005) or severe skin reactions (Nielsen et al. 2006). Despite listed limitations parecoxib is approved by European Medicines Agency n.d. to treat postoperative pain (http://www.ema.europa.eu).

In conclusion, results of our study provide novel mechanism of niflumic acid and parecoxib action in rat brain cortex independent from COX-2 inhibition. Through enzymatic inhibition of brain KYNA synthesis niflumic acid and parecoxib may be considered as a potential adjunct therapy in schizophrenia or cognitive decline treatment, what requires further investigations.

## Electronic Supplementary Material

Table S1 (DOCX 17 kb)

## Abbreviations

COX-2: cyclooxygenase-2
CRP: C-reactive protein
DA: dopamine
DFU: (5,5-dimethyl-3-(3-fluorophenyl)-4-(4-methylsulphonyl) phenyl-2((5)H)-furanone)
DMSO: dimethyl sulfoxide
GLU: glutamate
HPLC: high-performance liquid chromatography
IDO: indoleamine 2,3-dioxygenase
IL-1β: interleukin-1 beta
IL-2: interleukin-2
IL-6: interleukin-6
IL-10: interleukin-10
KAT: kynurenine aminotransferase
KYN: kynurenine
KYNA: kynurenic acid
LTP: long-term potentiation
MK-801: dizocilpine
NMDA: *N*-methyl-D-aspartate
PMP: 4′-deoxy-4′-aminopyridoxal-5′-phosphate
