# Supplementary material for: Influence of Cyclooxygenase-2 Inhibitors on Kynurenic Acid Production in Rat Brain in Vitro
Source: Neurotox Res. 2018 Sep 3;35(1):244–54. doi: 10.1007/s12640-018-9952-9 (PMC6313367; doi:10.1007/s12640-018-9952-9)
Supplement: Supplementary file 1 — (DOCX 17 kb) [file 12640_2018_9952_MOESM1_ESM.docx]

Influence of cyclooxygenase-2 inhibitors on kynurenic acid production in rat brain *in vitro*

Izabela Zakrocka^1^, Katarzyna M. Targowska-Duda^2^, Artur Wnorowski^2^,
Tomasz Kocki^1^, Krzysztof Jóźwiak^2^, Waldemar A. Turski^1^

^1^Department of Experimental and Clinical Pharmacology, Medical University of Lublin, Lublin, Poland

^2^Department of Biopharmacy, Medical University of Lublin, Lublin, Poland

Address for correspondence: Izabela Zakrocka, Department of Experimental and Clinical Pharmacology, Medical University of Lublin, Jaczewskiego 8b, 20-090 Lublin, Poland; e-mail: izabela.zakrocka@umlub.pl; phone: +48 81448 6453; fax: +48 81448 6451.

**Table S1** Residues involved in the binding of KYN, and COX-2 inhibitors, including niflumic acid and parecoxib to human KAT II active site. Residues interacting with KYN (PDB ID: 2R2N) as well as common residues for KYN and other ligands are shown in green.

| Compound | Binding site | Hydrogen Bonds | Salt bridge | HBonds with co-factor |
| --- | --- | --- | --- | --- |
| Kynurenine  PDB | Ile19 (A)  Arg20 (A)  Gly39 (A)  Leu40 (A)  Tyr74 (A)  Leu293 (A)  Tyr142 (B)  Ser143 (B)  Asn202 (B)  Tyr233 (B)  Phe355 (B)  Phe387 (B)  Arg399 (B) | Asn202  Tyr74  Ser142  Ser143 | Arg399 | No |
| PMP (Co-factor) | Gly39 (A)  Tyr74 (A)  Leu293 (A)  Gly116 (B)  Ser117 (B)  Gln118 (B)  Leu121 (B)  Tyr195 (B)  Val197 (B)  Asp230 (B)  Pro232 (B)  Ser260 (B)  Ser262 (B)  Arg270 (B) | Ser117  Gln118  Ser262  Arg270 | No | Not determined |
| Niflumic acid  orientation 1 | Ser17 (A)  Ile19 (A)  Arg20 (A)  Gly39 (A)  Leu40 (A)  Tyr74 (A)  Ser77 (A)  Leu293 (A)  Gln289 (A)  Tyr142 (B)  Ser143 (B)  Gly144 (B)  Asn202 (B)  Phe355 (B)  Arg399 (B) | Tyr142  3 H_2_O | No | No |
| Niflumic acid  orientation 2 | Ser17 (A)  Ile19 (A)  Arg20 (A)  Gly39 (A)  Leu40 (A)  Tyr74 (A)  Ser77 (A)  Leu293 (A)  Tyr142 (B)  Ser143 (B)  Gly144 (B)  Asn202 (B)  Phe355 (B)  Phe387 (B)  Arg399 (B) | Tyr142  Gly39 | No | Yes |
| Niflumic acid  orientation 3 | Ser17 (A)  Ile19 (A)  Arg20 (A)  Gly39 (A)  Leu40 (A)  Pro41 (A)  Tyr74 (A)  Ser77 (A)  Leu293 (A)  His294 (A)  Gln289 (A)  Tyr142 (B)  Ser143 (B)  Gly144 (B) | Tyr142  2 H_2_O | No | No |
| Niflumic acid  orientation 4 | Ile19 (A)  Arg20 (A)  Thr23 (A)  Gly38 (A)  Gly39 (A)  Leu40 (A)  Tyr74 (A)  Ser75 (A)  Leu293 (A)  Tyr142 (B)  Ser143 (B)  Asn202 (B)  Phe355 (B)  Arg399 (B) | 3 H_2_O | No | No |
| Parecoxib  orientation 1 | Ser17 (A)  Ile19 (A)  Arg20 (A)  Thr23 (A)  Gly38 (A)  Gly39 (A)  Leu40 (A)  Tyr74 (A)  Ser77 (A)  Gln289 (A)  Leu293 (A) His294 (A)  Tyr142 (B)  Ser143 (B)  Gly144 (B)  Asn202 (B)  Phe355 (B)  Phe387 (B)  Arg399 (B) | Arg399  Agr20  2 H_2_O | No | No |
| Parecoxib  orientation 2 | Ile19 (A)  Arg20 (A)  Thr23 (A)  Gly38 (A)  Gly39 (A)  Leu40 (A)  Tyr74 (A)  Ser75 (A)  Leu293 (A) His294 (A)  Tyr142 (B)  Ser143 (B)  Gly144 (B)  Asn202 (B)  Phe355 (B)  Leu382 (B)  Phe387 (B)  Arg399 (B) | Arg399  Asn202  2 H_2_O | No | Yes |
